# 

**Published:** 1871-10

**Authors:** 


### Contents of October Number.

### ORIGINAL DEPARTMENT.

ART. 1, Thesis—Excisions involving the joints of the upper extremity. Reports of illustrative cases. By W. W. Miner, class of 1870-71..... 81
ART. 2, Abstract of remarks on diseases of the Ear and their treatment. By Dr. Charles E. Rider, before the Rochester City Medical Society, September 4th, 1871.......................... 89
ART. 3, Abstract of the proceedings of the Buffalo Medical Association,... 94
ART. 4, Clinical Lectures upon Surgical cases in the Buffalo Hospital of the Sisters of Charity. By Prof. J. F. Miner. Reported by F. E. L. Brecht, member of the class of 1870-71........................ 97

### MISCELLANEOUS.

Varieties of the Pulse. By Prof. Chas. W. Chancellor, M. D. ..........102
The Treatment of Syphilis..........................................106
Cundurango.—How to treat Humbugs ..................................108

### CORRESPONDENCE.

Clinics in Bellevue. By C. H. Richmond, M. D........................112

### EDITORIAL DEPARTMENT.

Commencement of Lecture Terms in Medical Colleges, &c...............115
Cundurango at Less Price ...........................................116
Prize Essay on "Diseases of Children"...............................116
Introductory Lecture in the Buffalo Medical College,................117

BOOKS REVIEWED:

The Physicians Prescription Book,...................................117
Hand Book of Practical Obstetrics, including Anæsthesia. By John Tanner, M. D........................117
Essay on growths in the Larynx. By Morell Mackenzie, M. D. 117
The Physical Diagnosis of Brain Disease. By Reuben A. Vance, M. D..........................118

BOOKS AND PAMPHLETS RECEIVED........................................118
Skin Diseases.—The Students' Classification of Skin Diseases. By J. W. Southworth, M. D..........................119

---

THE BUFFALO

